# Addressing stigma in infectious disease outbreaks: a crucial step in pandemic preparedness

**DOI:** 10.3389/fpubh.2023.1303679

**Published:** 2023-12-22

**Authors:** Amy Paterson, Piero L. Olliaro, Amanda Rojek

**Affiliations:** ^1^Pandemic Sciences Institute, University of Oxford, Oxford, United Kingdom; ^2^Faculty of Health Sciences, University of Cape Town, Cape Town, South Africa

**Keywords:** infectious disease outbreaks, emerging infectious diseases, stigma, epidemic, pandemic preparedness

## Abstract

There is a complex interplay between infectious disease outbreaks and the stigmatization of affected persons and communities. Outbreaks are prone to precipitating stigma due to the fear, uncertainty, moralisation, and abatement of freedoms associated with many infectious diseases. In turn, this stigma hampers outbreak control efforts. Understanding this relationship is crucial to improving coordinated outbreak response. This requires valid and reliable methods for assessing stigma towards and within impacted communities. We propose adopting a cross-outbreak model for developing the necessary assessment tools. A stigma-informed approach must then be integrated into outbreak preparedness and response efforts to safeguard public health and promote inclusivity and compassion in future outbreaks.

Infectious disease outbreaks have long been accompanied by an insidious companion – stigma. The legacy of this association traces back to antiquity, encompassing leprosy (now known as Hansen’s disease), and reverberates through contemporary events such as the global outbreak of mpox (formerly known as monkeypox). Notably the renaming of both these diseases was driven by the imperative to disentangle them from this accompanying stigma (1, 2).

Stigma occurs when an individual or group is denied full social acceptance due to an attribute deemed discrediting by their community or society (3, 4). This umbrella term includes negative thoughts and feelings about affected individuals (i.e., prejudice) and negative treatment including exclusion (i.e., discrimination) (4–6). Stigma plays a prominent role in infectious disease outbreaks for a variety of reasons – the diseases are communicable, often unfamiliar, sometimes severe, and public health interventions can involve a lack of privacy and incursion on civil liberties.

Communities respond to outbreaks with spontaneous actions that can either precipitate or counteract the emergence of stigma. This ranges from exclusion or discrimination of individuals, as witnessed in the ostracization of people of Asian appearance in the initial stages of the COVID-19 outbreak (7), to mobilisation of support groups, such as those established by Ebola survivors in the Democratic Republic of the Congo (8), Sierra Leone (9), and Uganda (10). However, what often remains inadequately considered is the extent to which outbreak control activities might inadvertently precipitate stigma. This is despite the fact that outbreak control measures that could unintentionally foster stigma are seemingly ubiquitous.

Contact tracing, as conducted for COVID-19, often results in linear assignment of blame to affected individuals (11). Public health messaging that relies on instilling fear to drive behavior change can unintentionally foster stigma (12, 13). This is similarly true for messaging that moralizes diseases and health-related behavior (11, 12, 14). For instance, in Mexico, government communication on preventing Dengue has emphasised household cleanliness, leading those who get the disease to be perceived as unclean (15). Risk communication that singles out specific demographics or regions, as observed with COVID-19 (16), SARS (17), and mpox (18), may contribute to intersectional stigma for high risk groups. Hazmat suits and other personal protective equipment can create a sense of otherness for both patients and healthcare workers across outbreak settings (13, 19, 20). Additionally, the prohibition of traditional burial practices, a common measure for infection control in Ebola (21) and Nipah virus outbreaks (22), is viewed as disrespectful and stigmatizing in certain communities. A systematic review of the psychological impacts of enforced quarantine during COVID-19 reported enduring stigma as a recurring theme (23).

While this does not discredit the control measures *per se* – many outbreak interventions require a delicate dance of accepting some collateral harms and risks – these untoward consequences must be measured more robustly and reduced where possible. This is important for the well-being of affected populations and for optimising outbreak control.

Outbreak-associated stigma substantially impacts social, physical, and psychological well-being (24). This is particularly true when the stigma becomes internalized, also known as self-stigma, resulting in feelings of self-blame, guilt, and low self-confidence (25). These symptoms can reduce self-efficacy, motivation, and perceived control over negative events, including the spread of an outbreak, thereby diminishing the importance placed on preventive measures such as physical distancing (26).

Several studies have demonstrated that Ebola and COVID-19 stigma have strong associations with symptoms of depression, anxiety, insomnia, and post-traumatic stress disorder (PTSD) (27–31). Since these associated mental health conditions are often also stigmatized, this can add to the burden of stigma for affected individuals (32). A Poisson regression model, examining impact of the 2003 SARS epidemic, revealed an excess of older adult suicides at the peak of the epidemic, with the statistic not returning to baseline for at least 2 years (33). This trend has been observed before, during the 1918–19 influenza pandemic (34), and subsequently, with Ebola survivor suicide attempts six times higher than those of other community members in one cohort (35). In some cases, these mental health impacts have continued to affect survivors more than 20 years after the relevant outbreak (36).

This negative impact is not restricted to those who are diagnosed with an outbreak disease, but also those in close proximity with them (including family members and other community members), healthcare workers, and those thought to be associated with the disease (e.g., due to appearance or related symptoms) (37). Notably, in the Democratic Republic of the Congo, nearly half (46%) of individuals in cities and villages affected by the 2019 Ebola outbreak exhibited severe psychological distress symptoms seven months after the outbreak (37). In a population-based sample of adults in Michigan, the prevalence of depressive and anxiety symptoms was higher among respondents who perceived more COVID-19 stigma (31). Higher levels of outbreak-related anxiety in community members may consequently worsen bullying of those with the disease (38). These repercussions of stigma for affected populations perpetuate health and social inequities (24).

The adverse effects of stigma also impact outbreak control permeating every aspect of outbreak response (Figure 1). These insidious effects can be identified across geographical settings and diseases. For instance, during the Ebola outbreak in West Africa, contact tracers encountered community resistance and non-disclosure of contacts, hindering timely interventions (39). Similarly, in the COVID-19 pandemic, testing efforts suffered from denial, symptom concealment, and avoidance of testing facilities, leading to underestimation of cases and undetected spread (40, 41). As a further example of this, Ebola survivors from Sierra Leone reported they took an average of 3.36 days to seek treatment after symptom onset (41).

**Figure 1:**
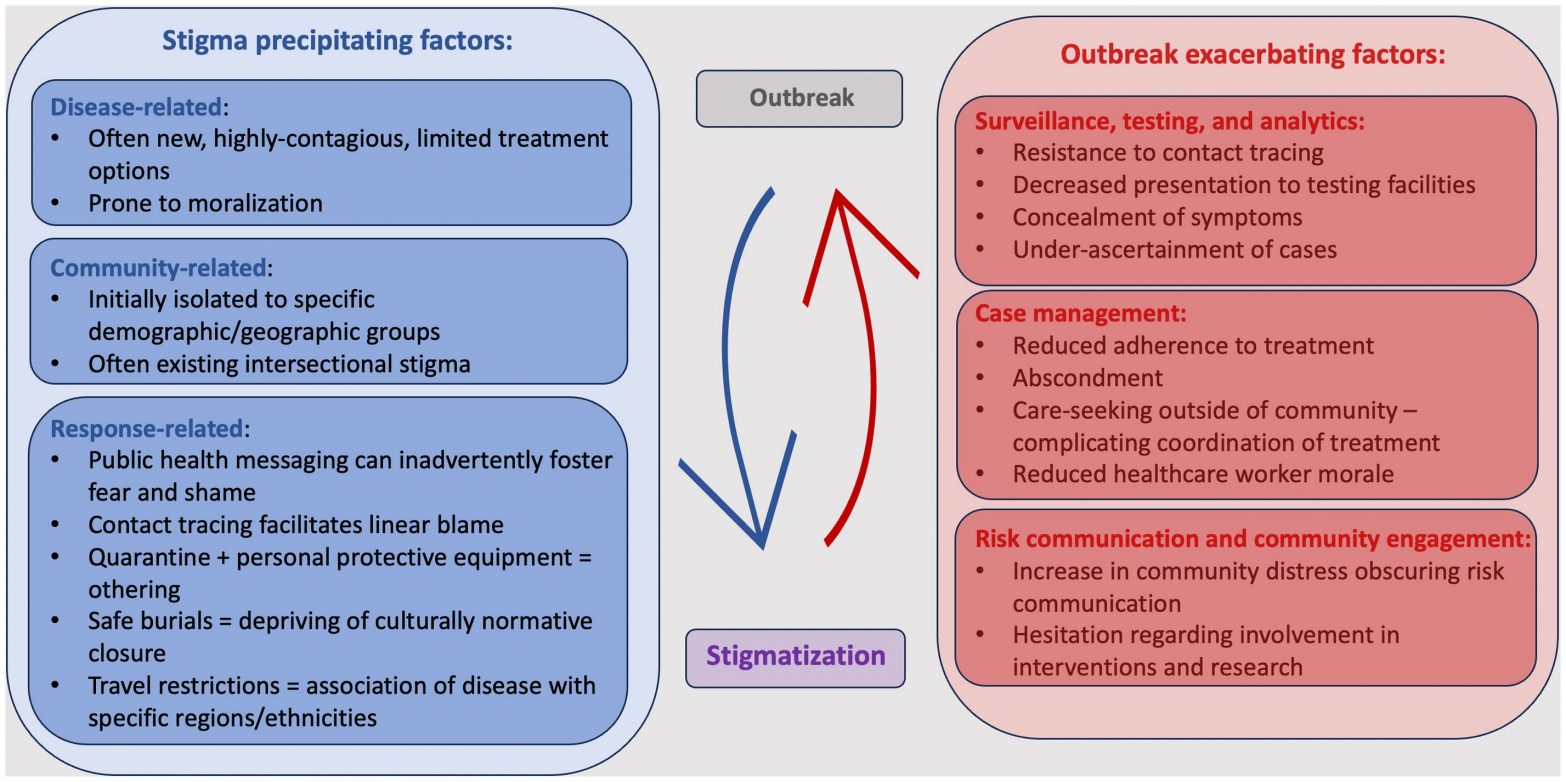
The interplay between stigma and infectious disease outbreaks.

In many outbreaks stigma poses a significant challenge to case management by reducing adherence to care and treatment (15, 21). Lack of trust in responders can also risk healthcare worker safety. A cross-sectional study of over 7,000 participants in 173 countries found that healthcare workers were at an increased risk of COVID-19 related bullying and harassment (42). In turn, healthcare worker fear and diminished morale may result in hesitation to treat individuals thought to have an outbreak disease (17, 21). This occurred during the SARS outbreak in Hong Kong, where occupants of a high risk residential complex reported being denied healthcare services at clinics (17).

Infection prevention faces hurdles with stigma fostering avoidance of preventative measures, vaccine hesitancy, and disease denialism. The recent mpox outbreak is an example of this, stigma related to the required disclosure of sexual preferences for vaccination has discouraged vaccine uptake in many countries (23). Epidemiological analytics suffer from under-ascertainment of cases when individuals do not disclose their infections. Risk communication is clouded by social anxieties and decreased interaction with the health system limits educational opportunities, as was found to be the case with Lassa fever in Nigeria (43) and Zika virus disease in Spain (44). Research which is scientifically robust and ethically valid may still fail due to scepticism and fear.

The HIV/AIDS pandemic provides compelling empirical evidence of how stigma can impact infectious disease detection, service uptake, and clinical outcomes (45, 46). For instance, recent data from UNAIDS reveals that addressing stigma and discrimination related to HIV could prevent 2.5 million new HIV infections and avert 1.7 million AIDS related deaths by 2025 (45).

This evidence underscores the need to prioritize addressing stigma in disease control efforts, and has spurred international and national commitments to address HIV stigma (46–48). Notably, the 2021 UN Political Declaration on HIV and AIDS (48) and Global AIDS Strategy (2021–2026) (47) introduced the 10-10-10 targets to achieve HIV control. These targets require countries to undertake reforms to ensure that less than 10% of people living with HIV and key populations will experience HIV-related or intersectional stigma (47, 48). Additionally, the targets aim to reduce structural discrimination such that less than 10% of countries have legal and policy frameworks that limit access to HIV-related services (47, 48).

As a result of these commitments, HIV stigma is now carefully monitored across a wide range of settings (49). Robust assessment tools have been developed for this purpose. For example, the People Living with HIV (PLHIV) stigma index evaluates the effect of HIV stigma on public health in a standardised manner globally. It has subsequently been used for public health and advocacy purposes in more than 100 countries with over 100,000 participants (50). A cross-culturally validated and widely used stigma measure for emerging disease outbreaks could similarly enhance our ability to reveal and mitigate stigma in emerging infectious disease outbreaks.

We advocate for rapid and repeated assessment of stigma as an equally important component of emerging outbreak response. This is critical not only to inform and evaluate strategies for stigma reduction, but also to provide reliable guidance to responders on how to limit the propagation of stigma (51). While outbreak-specific tools exist for assessing stigma (17, 30, 43, 52–58), this assessment currently tends to occur late in the course of an emerging outbreak, limiting the potential impact of this work. This delay is often due to the lead time required for the creation of new stigma assessment tools each time an outbreak occurs.

Cross-outbreak research on stigma could facilitate faster assessment of stigma and mitigation of stigma’s adverse effects on the mental health of affected individuals as well as outbreak control. This is because it allows us to begin creating and validating tools to assess and address stigma associated with future outbreaks, even before the specific details of the next outbreak are known. This approach adopts the concept of “disease X” as an exemplar – a placeholder name widely adopted in vaccine development and clinical research to represent an as yet unknown emerging pathogen that should be included in pandemic preparedness (59).

The development of cross-outbreak stigma research tools has pragmatic benefits: many of these diseases are rare, occur sporadically, and emerge at unexpected times and locations. While high impact, their duration is often brief. A stigma tool designed reactively to an outbreak is often too late to facilitate timely intervention, or of limited validity if developed quickly (60). Local outbreak response efforts usually do not have the capacity to create such a tool in resource-limited settings, or indeed amongst competing demands even in high-resource settings.

Importantly, the development of stigma research tools that can be used across emerging infectious diseases is also feasible. This is due to notable similarities in the manifestations of stigma across different settings and diseases (Figure 2) (61). Consequently, we propose adopting this cross-outbreak model to proactively design evidence-based outbreak stigma assessment and intervention tools with broad applicability. These tools can then be readily tailored to suit local contexts.

**Figure 2:**
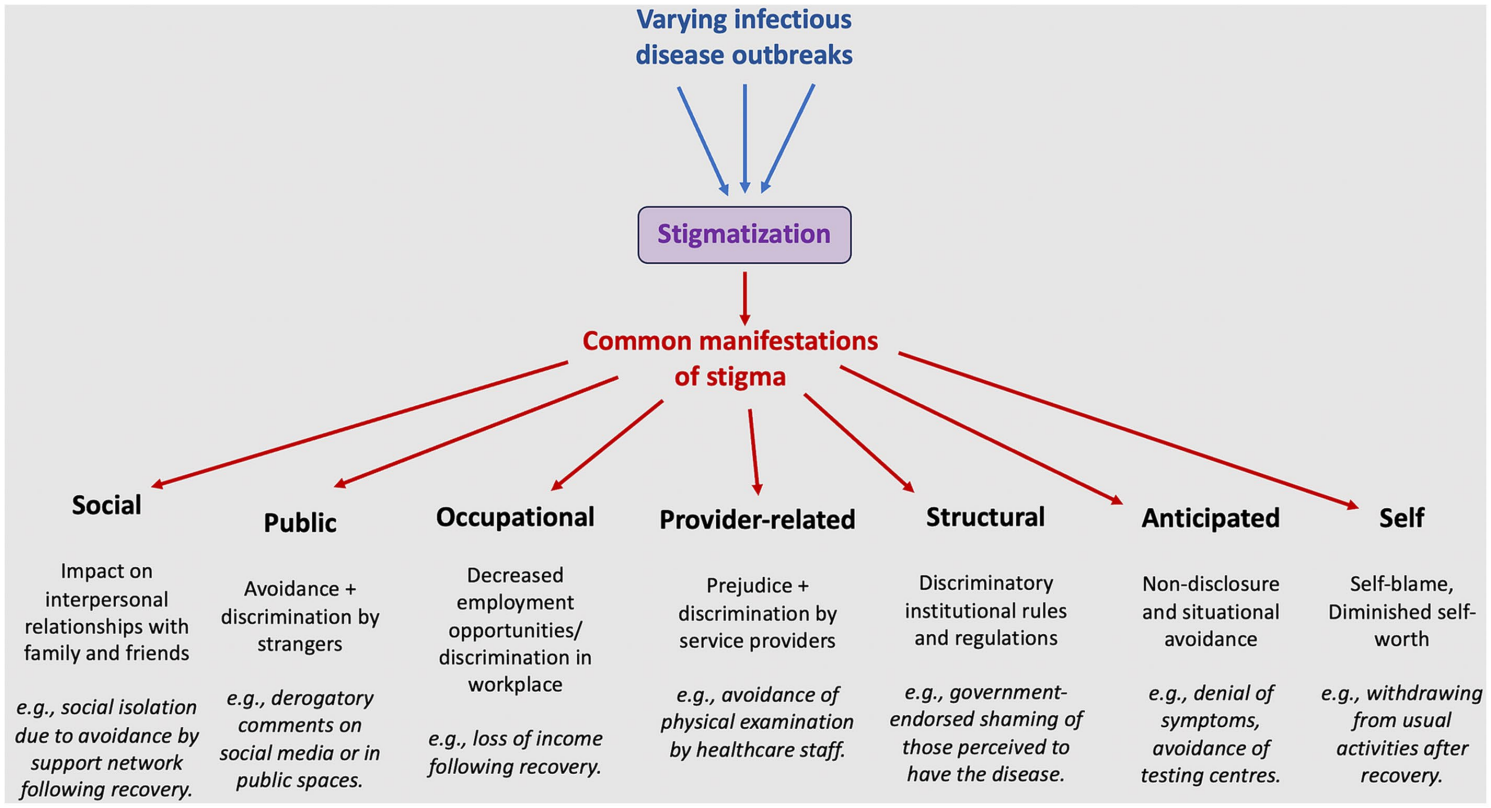
Commonalities in the manifestations of outbreak-associated stigma.

An approach that considers and intentionally minimises stigma should be embedded in outbreak preparedness and response efforts. It is imperative to ensure that stigma does not hinder our ability to safeguard public health, protect affected communities, and promote a culture of inclusivity and compassion in the face of future outbreaks.

## Data availability statement

The original contributions presented in the study are included in the article/supplementary material, further inquiries can be directed to the corresponding author.

## Author contributions

AP: Conceptualization, Writing – original draft, Writing – review & editing. PO: Supervision, Writing – review & editing. AR: Conceptualization, Supervision, Writing – review & editing.
